# Conformal QED in two-dimensional topological insulators

**DOI:** 10.1038/s41598-017-14635-y

**Published:** 2017-10-26

**Authors:** Natália Menezes, Giandomenico Palumbo, Cristiane Morais Smith

**Affiliations:** Institute for Theoretical Physics, Center for Extreme Matter and Emergent Phenomena, Utrecht University, Princetonplein 5, 3584 CC Utrecht, The Netherlands

## Abstract

It has been shown that local four-fermion interactions on the edges of two-dimensional time-reversal-invariant topological insulators give rise to a new non-Fermi-liquid phase, called helical Luttinger liquid (HLL). Here, we provide a first-principle derivation of this HLL based on the gauge-theory approach. We start by considering massless Dirac fermions confined on the one-dimensional boundary of the topological insulator and interacting through a three-dimensional quantum dynamical electromagnetic field. Within these assumptions, through a dimensional-reduction procedure, we derive the effective 1 + 1-dimensional interacting fermionic theory and reveal its underlying gauge theory. In the low-energy regime, the gauge theory that describes the edge states is given by a conformal quantum electrodynamics (CQED), which can be mapped exactly into a HLL with a Luttinger parameter and a renormalized Fermi velocity that depend on the value of the fine-structure constant *α*.

## Introduction

Topological insulators represent a large family of materials characterized by gapped bulks and metallic edge states. The topological quantum numbers associated to the bulk depend on the discrete symmetries of the microscopic Hamiltonians, such as time-reversal, particle-hole and chiral symmetries^1,2^. Further spatial (crystalline) symmetries have been proposed in order to extend the periodic table of topological free-fermion systems^3,4^, and more recently inversion symmetry has also gathered attention^5,6^. However, time-reversal-invariant topological insulators are certainly the most studied so far^7,8^. These time-reversal-invariant topological insulators were theoretically proposed to occur in two-dimensional models involving a strong spin-orbit interaction^9,10^, and were then experimentally observed in HgTe quantum wells^11^. The spin-orbit interaction locks the spin and the chirality together and produces counter-propagating edge currents, giving rise to the quantum spin Hall effect. These topologically protected edge modes are right-handed and left-handed Dirac modes that always come in pairs, in agreement with the time-reversal symmetry of the bulk. Their dynamics is consistently described by a 1 + 1-dimensional massless Dirac theory.

Moreover, it has been shown that local four-fermion interactions on the edge can transform the free-fermion phase into a new non-Fermi-liquid phase, called helical Luttinger liquid (HLL)^12,13^. In this picture, the strength of the interactions is encoded in the Luttinger parameter *K*, which depends on the value of the coupling constant *g* of the four-fermion term. Although many studies have pointed out for which values of *K* the interactions are relevant, it is still unclear how the constant *g* is related to the microscopic properties of the Dirac edge modes, such as their spin, electric charge, etc. The relevant open question is whether there is any fundamental way to derive the HLL from the universal properties of topological insulators.

**Figure 1:**
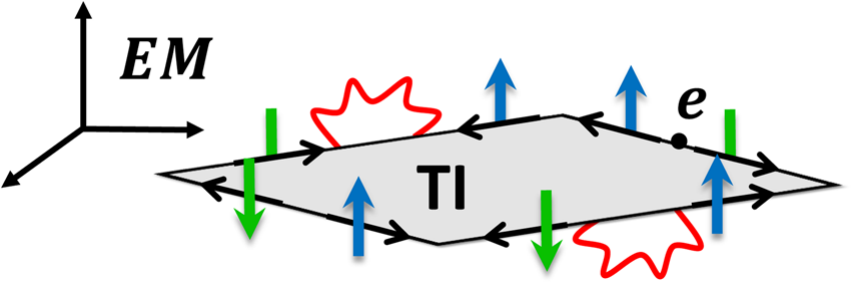
The red wavy lines represent the virtual photons that are free to propagate in all the three spatial dimensions, while the massless Dirac fermions with electric charge *e* are confined on the one-dimensional boundary of the topological insulator. The arrows at the edges indicate the propagation of the topologically protected right- and left-handed chiral modes.

The main goal of this paper is to provide an answer to this question. Firstly, we consider massless Dirac fermions constrained in one spatial dimension (the boundary), while the quantum excitations (i.e. the virtual photons) of the *U*(1) gauge field are free to propagate in all the three spatial dimensions that represent the physical space where the topological insulator is embedded, see Fig. 1. From this assumption, we derive the interacting fermionic theory for the edge states of two-dimensional (2D) time-reversal-invariant topological insulators. By using a Hubbard-Stratonovich transformation, we determine the effective 1 + 1-dimensional gauge theory that mediates the fermionic interaction, which is given by the sum of a conformal quantum electrodynamics (CQED)^14,15^ plus the 1 + 1-dimensional massless QED, also known as the Schwinger model^16,17^.

In this work, we focus on the CQED because describes a massless mode along the whole edge and is dominant in the low-energy regime. It also preserves the dimensionality of both, the electric charge and the gauge field of the 3 + 1-dimensional QED from which the CQED will be derived by using a dimensional reduction procedure. This method has been already used in studies of graphene^18,19^ and related 2D massive Dirac systems, such as silicene and transition metal dicalcogenides^20^, but to the best of our knowledge, it has not yet been employed in the description of one-dimensional systems, such as the edge currents of topological insulators. Notice that in ref.^21^, a projection of QED in 3 + 1-dimensions to a 1 + 1-brane was performed. However, a finite-size regulator was introduced to avoid ultraviolet divergences that appear when confining the system to one dimension. Therefore, the effective theory obtained is not scale invariant and cannot be conformal. In our approach, we found an explicit way to deal with the divergences, such that we obtain the CQED without any regulator. Furthermore, by integrating out the CQED gauge field in the corresponding partition function, we find that this gauge theory gives rise to a 1 + 1-dimensional Thirring model^22^. We then demonstrate that the bosonized version of the interacting-fermion Hamiltonian describes exactly a HLL with a Luttinger parameter *K* and a renormalized Fermi velocity that depend on the value of the fine-structure constant *α*.

## Conformal QED on the boundary of topological insulators

We start by considering two-dimensional time-reversal invariant topological insulators in class AII^2^. They have a gapped bulk and topologically protected Dirac edge modes. These systems realize the quantum spin Hall effect, i.e. the chirality of the Dirac edge modes is locked to the spin, which is preserved due to the time-reversal symmetry. Thus, the dynamics of the edge modes can be described by a 1 + 1-dimensional massless Dirac theory with a two-component Dirac spinor *ψ* = (*ψ*_*R*_, *ψ*_*L*_)^*T*^, where *ψ*_*R*_ and *ψ*_*L*_ are the right-handed spin-up and left-handed spin-down chiral modes, respectively. It was theoretically proposed in refs^12,13^ and experimentally confirmed in ref.^23^ that these topological insulators can support HLLs on the boundary due to the presence of unavoidable electron-electron interactions. These non-Fermi liquid phases fully preserve the time-reversal symmetry and are formally described by the free Dirac theory plus suitable four-fermion interactions. We now show that this model and the corresponding HLL can be derived from a gauge theory by simply assuming that the electrically charged propagating Dirac fermions on the edge interact through a quantum dynamical electromagnetic field *A*_*ρ*_. The essential point of our approach is that the massless Dirac fermions are confined on the one-dimensional boundary, whereas the quantum excitations (i.e. photons) of the electromagnetic field are free to propagate in all the three spatial dimensions, as shown in Fig. 1. The corresponding covariant QED action reads

1 $${S}_{{\rm{QED}}}[{A}_{\rho },\bar{\psi },\psi ]=i\hslash \,\int \,{d}^{2}r\,\bar{\psi }{\gamma }^{\mu }{\partial }_{\mu }\psi -\int \,{d}^{4}r\,(\frac{{\varepsilon }_{0}c}{4}\,{F}_{\rho \beta }{F}^{\rho \beta }+e{j}_{3+1}^{\rho }{A}_{\rho }),$$

where *d* ^2^ *r* = *vdxdt* and *d* ^4^ *r* = *cdxdydzdt*, with *v* and *c* the Fermi velocity and the speed of light, respectively. *ħ* is the Planck constant divided by 2*π*, *e* is the electric charge carried by each fermion, *ε*_0_ is the vacuum dielectric constant, *γ* ^*μ*^ are 2 × 2 Dirac matrices with *μ* = 0, 1 and {*γ* ^*ν*^, *γ* ^*μ*^} = 2*g* ^*μν*^, where *g* ^*μν*^ = diag(1, −1, −1), *F*_*ρβ*_ = ∂_*ρ*_ *A*_*β*_ − ∂_*β*_ *A*_*ρ*_ is the field-strength tensor, ${j}_{3+1}^{\rho }=\bar{\psi }{\gamma }^{\rho }\psi$, and $\bar{\psi }={\psi }^{\dagger }{\gamma }^{0}$ with *ρ*, *β* = 0, 1, 2, 3. The effective interaction felt by the massless Dirac fermions due to the gauge field can be obtained by integrating out the *A*_*ρ*_-field in the partition function ${\mathscr{Z}}$, i.e.

2 $${\mathscr{Z}}=\int \,{\mathscr{D}}\bar{\psi }\,\int \,{\mathscr{D}}\psi \,\int \,{\mathscr{D}}{A}_{\rho }\,\exp \,(\frac{i}{\hslash }\,{S}_{{\rm{QED}}})=\int \,{\mathscr{D}}\bar{\psi }\,\int \,{\mathscr{D}}\psi \,\exp \,(\frac{i}{\hslash }\,{S}_{{\rm{eff}}}[\bar{\psi },\psi ]),$$

where *S*_eff_ = *S*_*D*_ + *S*_int_ is the effective action, with *S*_*D*_ the free Dirac action, given by the first term in Eq. (1), and *S*_int_ the interaction term, given by

3 $${S}_{{\rm{int}}}=-\frac{{e}^{2}}{2{\varepsilon }_{0}c}\,\int \,{d}^{4}r{d}^{4}r^{\prime} {j}_{3+1}^{\rho }(r)\frac{1}{(-\square )}{j}_{\rho }^{3+1}(r^{\prime} ),$$

where we performed the Wick rotation and $\square$ is the d’Alembertian operator in the Euclidean space. Now, by imposing a constraint on the matter current,

4 $${j}_{3+1}^{\rho }(t,x,y,z)={j}_{1+1}^{\mu }(t,x)\,\delta (y)\,\delta (z),$$

we create the dimensional mismatch between the Dirac fermions and the virtual photons, preserving the 3 + 1 spacetime dimensionality of the electromagnetic field. Hence, by inserting Eq. (4) into Eq. (3), we get

5 $${S}_{{\rm{int}}}=-\frac{{e}^{2}}{2{\varepsilon }_{0}c}\,\int \,{d}^{2}r{d}^{2}r^{\prime} {j}_{1+1}^{\mu }(r)\,{[\frac{1}{(-\square )}]}_{\ast \ast }\,{j}_{\mu }^{1+1}(r^{\prime} ),$$

where the symbol $\ast \ast$ means that we need to evaluate the Green’s function at *y* = *y*′ = 0 and *z* = *z*′ = 0. To evaluate Eq. (5), we first write the Fourier transform of the Green’s function

6 $$\frac{1}{(-\square )}=-\square \,\int \,\frac{{d}^{4}k}{{\mathrm{(2}\pi )}^{4}}\frac{{e}^{ik\cdot (r-r^{\prime} )}}{{({k}^{2})}^{2}},$$

where $\square ={\partial }_{t}^{2}+{\partial }_{x}^{2}+{\partial }_{y}^{2}+{\partial }_{z}^{2}$ acts on the coordinates. We integrate over the momenta *k* and then impose the above constraints on the coordinates, to eventually find (see Supplemental Material for details)

7 $${[\frac{1}{(-\square )}]}_{\ast \ast }=\frac{1}{2\pi }\delta (x-x^{\prime} )\,\delta (t-t^{\prime} )+\frac{1}{4{\pi }^{2}}\frac{1}{{\square }_{1+1}},$$

where *δ*(*x* − *x*′) and *δ*(*t* − *t*′) are two Dirac delta functions and ${\square }_{1+1}$ is the d’Alembertian in 1 + 1 dimensions. Notice that in refs^21,24^, a finite-size regulator for the Dirac delta function in Eq. (4) was introduced. This result agrees with ours in the limit when the finite-size regulator is removed.

The replacement of the terms in Eq. (7) in the effective interaction (5) leads to

8 $${S}_{{\rm{int}}}=-\frac{{e}^{2}}{4\pi {\varepsilon }_{0}c}\,\int \,{d}^{2}r{j}_{1+1}^{\mu }(r){j}_{\mu }^{1+1}(r)-\frac{{e}^{2}}{8{\pi }^{2}{\varepsilon }_{0}c}\,\int \,{d}^{2}r^{\prime} {d}^{2}r{j}_{1+1}^{\mu }(r)\frac{1}{{\square }_{1+1}}{j}_{\mu }^{1+1}(r^{\prime} ).$$

By using a Hubbard-Stratonovich transformation, we rewrite individually each Gaussian-type interaction in Eq. (8) in terms of new and independent auxiliary (1 + 1)D gauge fields ${{\mathscr{A}}}_{\mu }^{a}$ (with *a* = 1, 2), and obtain

9 $$S[{{\mathscr{A}}}_{\mu }^{a},\bar{\psi },\psi ]=\int \,{d}^{2}r\,(i\hslash \bar{\psi }{\gamma }^{\mu }{\partial }_{\mu }\psi -e{j}_{1+1}^{\mu }{{\mathscr{A}}}_{\mu }^{1}-\tfrac{\pi {\varepsilon }_{0}c}{2}{F}_{\mu \nu }^{1}\tfrac{1}{{\square }_{1+1}}{F}_{1}^{\mu \nu }-\bar{e}{j}_{1+1}^{\mu }{{\mathscr{A}}}_{\mu }^{2}-{\pi }^{2}{\varepsilon }_{0}c\,{F}_{\mu \nu }^{2}{F}_{2}^{\mu \nu }),$$

which replaces the action (1) and represents the main result of this work. By integrating out the ${{\mathscr{A}}}_{\mu }^{a}$-fields in Eq. (9) one obtains, besides the free Dirac action, exactly the interacting terms given by Eq. (8) (see Sup. Mat. for details). From our result (9) we can derive two well-known exactly solvable models in 1 + 1-dimensions: by integrating out the ${{\mathscr{A}}}_{\mu }^{1}$-field, we obtain the Thirring model^22^, whereas the Lagrangian for the ${{\mathscr{A}}}_{\mu }^{2}$-field can be identified with the Schwinger model^16,25^. The pseudo-differential operator in the kinetic term of the ${{\mathscr{A}}}_{\mu }^{1}$-field determines its dimensionality, such that the coupling constant *e* remains dimensionless, while $\bar{e}=e{\rm{\Lambda }}$ is a dimensionful bare constant and Λ has a mass dimension (see sup. mat. for more details).

It is known that the Schwinger-Thirring model leads to a massless and a massive bosonic mode^26,27^. However, in the low-energy limit, i.e. $k\ll ev{\rm{\Lambda }}$, only the former describes a propagating mode along the whole edge. The massive mode is localized and may be accessed only at higher values of the energy. Moreover, this massless bosonic mode reveals the critical – zero mass – nature of the original fermion. From now on, by focusing on the low-energy regime, we proceed our analysis by neglecting the contribution from the massive ${{\mathscr{A}}}_{\mu }^{2}$-field.

We want to emphasize that the dimensional reduction procedure performed here has been already employed in the study of two-dimensional materials, such as graphene. In this case, the corresponding effective field theory is the so-called Pseudo QED (PQED)^28,29^, i.e., a (2 + 1)D QED with higher-order derivatives in the Maxwell term (see Table 1). When electrons are confined in (1 + 1)D, the non-local (higher derivative) Maxwell term of the effective theory in Eq. (9) leads to a conformal theory when *c* = *v* ^14,15^. Importantly, both time-reversal and conformal symmetries are relevant in the identification of the right interacting phase of the topological insulator in the low-energy regime. Thus, because the boundary of a 2D non-interacting topological insulator is described by a free conformal field theory defined in terms of a 1D Dirac theory, we will consider the conformal fixed point (*c* = *v*) even for the interacting phase by deriving the corresponding HLL in the following section. This CQED shares some properties with PQED. In fact, in both theories the electric charge *e* is a dimensionless parameter, as in usual (3 + 1)D QED. The fact that the coupling constant remains dimensionless makes perturbative studies more reliable. Moreover, just like in the Luttinger-liquid case, in PQED and in CQED the excitations are collective modes and there are no quasi-particles because the Green’s function has branch cuts instead of poles^24^.

**Table 1:** The bosonic sector of the QED, PQED and CQED in the second column for *ε*_0_ = *c* = 1.

U(1) gauge theories	Bosonic Lagrangians
1 + 1 CQED	$-\tfrac{\pi }{2}{F}_{\mu \nu }\tfrac{1}{{\square }_{1+1}}{F}^{\mu \nu }$
2 + 1 PQED	$-\tfrac{1}{2}{F}_{\mu \nu }\tfrac{1}{\sqrt{{\square }_{2+1}}}{F}^{\mu \nu }$
3 + 1 QED	$-\tfrac{1}{4}{F}_{\mu \nu }{F}^{\mu \nu }$

In lower dimensions, the Maxwell theory is replaced by suitable versions that contains pseudo-operators, i.e. (∂^2^)^−*η*^ with *η* = 1 or 1/2, to adjust and preserve the dimensionality of the coupling constant [*e*] = 1. This means that QED, PQED and CQED are renormalizable theories.

## Thirring model and helical Luttinger liquid

Here, we derive in a straightforward way the HLL from our effective field-theory model. The fermionic kinematical term in Eq. (1), together with the local interaction term in Eq. (8), allow us to write the purely effective fermionic action

10 $${S}_{1+1}^{{\rm{eff}}}=\int \,{d}^{2}r\,[i\hslash \bar{\psi }{\gamma }^{\mu }{\partial }_{\mu }\psi -g{(\bar{\psi }{\gamma }^{\mu }\psi )}^{2}],$$

which can be recognized as the massless Thirring model^22^, with the coupling constant *g* = *e* ^2^/4*πε*_0_ *c*. The corresponding Hamiltonian is then calculated by employing a Legendre transformation,

11 $${H}_{{\rm{eff}}}=v\,\int \,dx\,[i\hslash ({\psi }_{R}^{\dagger }{\partial }_{x}{\psi }_{R}-{\psi }_{L}^{\dagger }{\partial }_{x}{\psi }_{L})+\frac{{e}^{2}}{\pi {\varepsilon }_{0}c}{\psi }_{R}^{\dagger }{\psi }_{R}{\psi }_{L}^{\dagger }{\psi }_{L}],$$

where the interaction term is nothing but the forward scattering, and we have used the chiral basis with *ψ* = (*ψ*_*R*_, *ψ*_*L*_)^*T*^, with the fermion operators satisfying usual anti-commutation relations. The bosonization of Eq. (11) is straightforward^30^, and we obtain

12 $${H}_{{\rm{eff}}}^{{\rm{bos}}}=\tilde{v}\,\int \,dx\,[\frac{1}{K}{({\partial }_{x}\phi )}^{2}+K{({\partial }_{x}\theta )}^{2}],$$

which is the HLL Hamiltonian, with the scalar fields $\phi =({\varphi }_{R}+{\varphi }_{L})/\sqrt{2}$ and $\theta =({\varphi }_{R}-{\varphi }_{L})/\sqrt{2}$. Here, the bosonization rules read

13 $${\psi }_{R}=\frac{1}{\sqrt{2\pi }}\,{e}^{-i\sqrt{4\pi }{\varphi }_{R}},\quad {\psi }_{L}=\frac{1}{\sqrt{2\pi }}\,{e}^{i\sqrt{4\pi }{\varphi }_{L}},$$

with the Luttinger parameter *K* and the renormalized velocity $\tilde{v}$ respectively given by

14 $$K=\sqrt{(1-\frac{2\alpha }{\pi })\,{(1+\frac{2\alpha }{\pi })}^{-1}},$$

15 $$\tilde{v}=\hslash v\sqrt{1-\frac{4{\alpha }^{2}}{{\pi }^{2}}},$$

where *α* ≡ *e* ^2^/4*πħεv* is a measure of the strength of the electron-electron interaction, also known as the fine-structure constant. Because *α* is an observable that depends on the material, i.e. on the dielectric constant of the medium, *ε* = *ε*_*r*_ *ε*_0_ and *v* is the velocity of the fermions when they propagate in this material. Thus, due to gauge principle and to the projection from QED to CQED, we have been able to derive the HLL on the boundary of the topological insulator. Moreover, we have determined the value of the Luttinger parameter and the renormalized velocity, which depend, in our framework, only on the generic properties of the Dirac modes, i.e. the value of their electric charge, the Fermi velocity and the dielectric constant by means of the fine-structure constant *α*.

## Luttinger-parameter discussion

The parameter *K* in the HLL defines different regimes of the interaction, which changes from repulsive (*K* < 1), passing through non-interacting (*K* = 1), to attractive (*K* > 1)^31^. Nonetheless, how this parameter relates to fundamental properties of the materials was still unclear. In refs^32,33^, a formula that connects *K* with *α* is derived by employing perturbation theory with either the Kondo or the backscattering interaction. Here, we have presented a gauge-principle derivation of the Luttinger parameter, which is found to depend on the strength of the electron-electron interaction *α*.

Now, we compare our results with a prior theoretical prediction proposed in refs^32,33^, $K={[1+(8\alpha /\pi )\mathrm{ln}(d/\ell )]}^{-\mathrm{1/2}}$. Here, *d* is the distance from the quantum wells to a closeby metallic gate, and $\ell$ acts as a cutoff for short distances. This dependence of the parameter *K* on *α* was obtained at the level of perturbation theory on the HLL Hamiltonian, i.e. additional interaction terms had to be taken into account, such as the Kondo or the backscattering interaction. Although our approach is non-perturbative, there are implicit approximations based on the theoretical description of the edge states in terms of QED. The presence of metallic gates in realistic experiments, for instance, could have crucial influence on the field lines of the virtual photons and would modify the effective action in a non-trivial way. Using the values of the parameters reported experimentally for HgTe quantum wells, *v* ≈ 5.5 × 10^5^ m/s^11,34^, *ε*_*r*_ = 15^33,35^, *d* = 150 nm and $\ell =\,{\rm{\max }}\,\mathrm{\{30},12\}\,{\rm{nm}}$ ^23^, the authors in ref.^33^ find *K* ≈ 0.8. Within our model, which depends only on *α*, we obtain *K* ≈ 0.84.

Notice that our approach does not involve the backscattering term, which induces further corrections to the parameter *K*, as seen in the case of InAs/GaSb quantum wells^23^. This implies that our theoretical prediction applies to materials that have weak backscattering and high Fermi velocities, such as HgTe^36^. Nevertheless, the backscattering term can be obtained within our approach upon considering the *massive* Thirring model. The corresponding bosonization is discussed in the supplemental material with the Klein factors defined as in ref.^37^. Other possible 2D topological insulators that would be good candidates to test our theoretical proposal are plumbene monolayers^38^ and germanene films^39^. The Fermi velocity in these materials has the same order of magnitude as that in HgTe, indicating that backscattering might not be so relevant.

**Figure 2:**
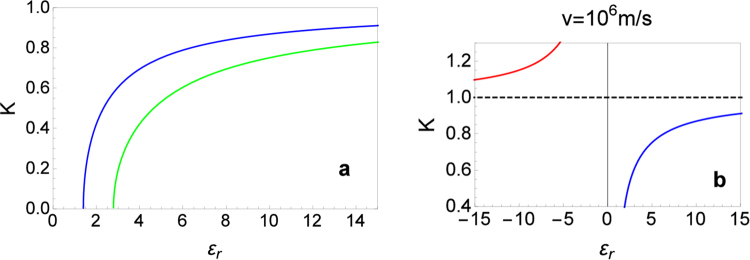
Luttinger parameter *K* dependence on the dielectric constant *ε*_*r*_ for fixed values of the Fermi velocity *v*. (**a**) The blue (black) and green (grey) curves are for *v* = 10^6^ m/s and *v* = 5 × 10^5^ m/s, respectively, and they indicate that for sufficiently large values of *ε*_*r*_, the system becomes non-interacting (*K* = 1), while for smaller values of *ε*_*r*_ the interaction is repulsive (*K* < 1). (**b**) A proposal to obtain attractive interactions *K* > 1 by changing the sign of the dielectric constant (red/grey curve) for a sample with *v* = 10^6^ m/s.

Furthermore, we show how to tune *K* in order to obtain different regimes of interaction. From Eq. (14), we notice that to change *K* we can either change *v* or the dielectric constant of the medium. In Fig. 2a, we depict the dependence of *K* on the dielectric constant *ε*_*r*_ in the range [1–15], for a fixed velocity *v* = 10^6^ m/s. In the asymptotic limit where *ε*_*r*_ → ∞ (meaning that we are considering very large values of the dielectric constant, not a mathematical infinity), it would be possible to reach the value of *K* = 1. For smaller velocities *v*, the minimum value of the dielectric constant for which *K* becomes real increases, i.e., for *v* = 5 × 10^5^ m/s, e.g., ${\varepsilon }_{r}^{{\rm{\min }}}\approx 2.7$, instead of ${\varepsilon }_{r}^{{\rm{\min }}}\approx 1.4$ for *v* = 10^6^ m/s. On the other hand, if we consider negative values of the dielectric constant by placing the topological insulator on top of a meta-material, then it is possible to switch from repulsive to attractive interactions, i.e., $K(x)\to K(-\,|x|)=\sqrt{(1+|x|)/(1-|x|)}$ with *x* = 2*α*/*π*. We illustrate this situation in Fig. 2b. The dielectric constant of the medium here plays the same role of Feshbach resonances in ultracold atoms, which allow to tune the interaction parameter from the repulsive to the attractive regime^40^.

## Conclusions

In this paper, we derived a gauge theory on the boundary of two-dimensional time-reversal-invariant topological insulators. Our starting point was to assume that the interactions between the charged one-dimensional Dirac fermions at the edge are mediated by a quantum dynamical electromagnetic field, where the virtual photons are free to propagate in all the three spatial dimensions. By implementing a dimensional-reduction procedure, we derived the corresponding CQED, which describes the HLL. We emphasize that our approach is non-perturbative, and has a more vast applicability in condensed-matter physics. Indeed, the one-dimensional effective theory derived here also works in the case of nanowires deposited on a substrate, in which the HLL phase can be easily obtained^41,42^, as done for topological insulators.

In our work, we provide, to the best of our knowledge, a field-theory derivation of the Thirring model, which opens the path to the manipulation of the Luttinger parameter *K* by modifying the dielectric constant of the substrate on which the one-dimensional system might be deposited. Interestingly, we find that upon the use of a meta-material as a substrate, it is possible to change the interactions from repulsive into attractive. These results might have profound implications for transport properties in nanostructures in particular, and nanotechnology in general.

## Electronic supplementary material

Supplemental Material
