# Supplementary material for: Conformal QED in two-dimensional topological insulators
Source: Sci Rep. 2017 Oct 26;7:14175. doi: 10.1038/s41598-017-14635-y (PMC5658404; doi:10.1038/s41598-017-14635-y)
Supplement: Supplementary file 1 — Supplemental Material [file 41598_2017_14635_MOESM1_ESM.pdf]

Supplemental Material:
Conformal QED in two-dimensional topological insulators

Natália Menezes[1], Giandomenico Palumbo[1] and Cristiane Morais Smith[1]
[1]*Institute for Theoretical Physics, Center for Extreme Matter and Emergent Phenomena, Utrecht University, Princetonplein 5, 3584 CC Utrecht, the Netherlands*
(Dated: September 15, 2017)

I. DETAILS OF THE CALCULATION ON THE PROJECTION FROM QED IN (3+1)D TO (1+1)D

Here, we show the detailed calculation starting from Eq. (5) to obtain Eq. (6) in the main text. The Fourier transform of the photon propagator reads

$$\frac{1}{(-\Box)} = \int \frac{d^4k}{(2\pi)^4} \frac{e^{ik\cdot(r-r')}}{k^2}, \tag{S1}$$

where $k^2 = k_x^2 + k_y^2 + k_z^2 + \omega^2$. First, we apply the constraint only on the z-component ($z = z' = 0$) and integrate Eq. (S1) over k_z to obtain

$$\left[\frac{1}{(-\Box)}\right]_* = \frac{1}{2}\int \frac{d^3k}{(2\pi)^3} \frac{e^{ik(r-r')}}{\sqrt{k^2}}, \tag{S2}$$

which is the known result of PQED [1]. The symbol $*$ means that we already imposed one of the constraints in the interaction term. Now, if one tries to follow the same steps and integrates over k_y, after applying the constraints on the y-component ($y = y' = 0$), the integral does not converge unless a cutoff is introduced. However, since our goal is to derive a conformal theory, we do not intend to introduce a new scaling in the theory by means of a cutoff.

We present an alternative way to solve this problem by rewriting Eq. (S2) as

$$\left[\frac{1}{(-\Box)}\right]_* = -\frac{\Box}{2}\int \frac{d^3k}{(2\pi)^3} \frac{e^{ik(r-r')}}{(k^2)^{3/2}} = -\frac{\Box}{4\pi}\int \frac{d^2k}{(2\pi)^2} e^{ik_x(x-x')+i\omega(t-t')} \int_{-\infty}^{\infty} dk_y \frac{e^{ik_y(y-y')}}{\left(k_x^2+k_y^2+\omega^2\right)^{3/2}}, \tag{S3}$$

where $\Box = \partial_t^2 + \partial_x^2 + \partial_y^2 + \partial_z^2$ is a differential operator that acts on the coordinates. The exponential in k_y can be expanded as

$$e^{ik_y(y-y')} = \sum_{n=0}^{\infty} \frac{i^n k_y^n (y-y')^n}{n!} = 1 + \sum_{n=1}^{\infty} \frac{i^n k_y^n (y-y')^n}{n!}. \tag{S4}$$

We split the contributions for $n = 0$ and $n > 0$ in the summation of Eq. (S4) to show explicitly how the contact interaction emerges.

Replacing Eq. (S4) into Eq. (S3) and focusing on the integral over k_y, we find

$$\int_{-\infty}^{\infty} dk_y \frac{1}{\left(k_x^2+k_y^2+\omega^2\right)^{3/2}} \left(1 + \sum_{n=1}^{\infty} \frac{i^n k_y^n (y-y')^n}{n!}\right) = \frac{2}{k_x^2+\omega^2} + \sum_{n=1}^{\infty} \frac{i^n (y-y')^n}{n!} \frac{[1+(-1)^n]\Gamma\left(1-\frac{n}{2}\right)\Gamma\left(\frac{n+1}{2}\right)}{\sqrt{\pi}\left(k_x^2+\omega^2\right)^{1-\frac{n}{2}}}, \tag{S5}$$

where the sum is only valid for even values of n, and for $n = 2$ the Gamma function has a pole. Fortunately, we show later that this pole cancels when one integrates further.

By replacing Eq. (S5) into Eq. (S3), we find

$$\left[\frac{1}{(-\Box)}\right]_* = -\frac{\Box}{4\pi}\int \frac{d^2k}{(2\pi)^2} e^{ik_x(x-x')+i\omega(t-t')} \left\{\frac{2}{k_x^2+\omega^2} + \sum_{n=1}^{\infty} \frac{i^n (y-y')^n}{n!} \frac{[1+(-1)^n]\Gamma\left(1-\frac{n}{2}\right)\Gamma\left(\frac{n+1}{2}\right)}{\sqrt{\pi}\left(k_x^2+\omega^2\right)^{1-\frac{n}{2}}}\right\}, \tag{S6}$$

and now we can apply the derivatives to the remaining functions. The first term of Eq. (S6) generates the local interaction, i.e.

$$-\frac{\Box}{2\pi}\int \frac{d\omega}{2\pi}\int \frac{dk_x}{2\pi} \frac{e^{ik_x(x-x')+i\omega(t-t')}}{k_x^2+\omega^2} = \frac{1}{2\pi}\delta(x-x')\delta(t-t'), \tag{S7}$$

which appears due to the first contribution of the expansion of Eq. (S4). The result obtained in Eq. (S7) does not depend on whether we consider or not the constraint on the y-component. However, this is not the case for the second term of Eq. (S6), which gives

$$
\begin{aligned}
&-\frac{1}{2\pi}\sum_{n \text{ even}}^{\infty}\frac{i^n\Gamma\left(1-\frac{n}{2}\right)\Gamma\left(\frac{n+1}{2}\right)}{\sqrt{\pi}(n!)}\Box\left[(y-y')^n\int\frac{d^2k}{(2\pi)^2}\frac{e^{ik_x(x-x')+i\omega(t-t')}}{\left(k_x^2+\omega^2\right)^{1-\frac{n}{2}}}\right]\\
&=-\frac{1}{2\pi}\sum_{n \text{ even}}^{\infty}\frac{i^n\Gamma\left(1-\frac{n}{2}\right)\Gamma\left(\frac{n+1}{2}\right)}{\sqrt{\pi}(n!)}\left[n(n-1)(y-y')^{n-2}\int\frac{d^2k}{(2\pi)^2}\frac{e^{ik_x(x-x')+i\omega(t-t')}}{\left(k_x^2+\omega^2\right)^{1-\frac{n}{2}}}\right]\\
&+\frac{1}{2\pi}\sum_{n \text{ even}}^{\infty}\frac{i^n(-n/2)\Gamma\left(-\frac{n}{2}\right)\Gamma\left(\frac{n+1}{2}\right)}{\sqrt{\pi}(n!)}\left[(y-y')^{n-2}\int\frac{d^2k}{(2\pi)^2}\frac{e^{ik_x(x-x')+i\omega(t-t')}}{\left(k_x^2+\omega^2\right)^{-\frac{n}{2}}}\right].
\end{aligned}
\tag{S8}
$$

Integrating over ω for both terms in Eq. (S8), we find

$$
\begin{aligned}
&\frac{1}{(2\pi)^2}\int_{-\infty}^{\infty}dk_x e^{ik_x(x-x')}\int_{-\infty}^{\infty}d\omega\frac{e^{i\omega(t-t')}}{\left(k_x^2+\omega^2\right)^{1-\frac{n}{2}}}\\
&=\frac{2^{(1+n)/2}\sqrt{\pi}}{(2\pi)^2\Gamma\left(1-\frac{n}{2}\right)}\int_{-\infty}^{\infty}dk_x e^{ik_x(x-x')}|t-t'|^{(1-n)/2}(k_x^2)^{\frac{n-1}{4}}K_{\frac{n-1}{2}}\left(|t-t'|\sqrt{k_x^2}\right),
\end{aligned}
\tag{S9}
$$

and

$$
\begin{aligned}
&\frac{1}{(2\pi)^2}\int_{-\infty}^{\infty}dk_x e^{ik_x(x-x')}\int_{-\infty}^{\infty}d\omega\frac{e^{i\omega(t-t')}}{\left(k_x^2+\omega^2\right)^{-\frac{n}{2}}}\\
&=\frac{2^{(3+n)/2}\sqrt{\pi}}{(2\pi)^2\Gamma\left(-\frac{n}{2}\right)}\int_{-\infty}^{\infty}dk_x e^{ik_x(x-x')}|t-t'|^{-(1+n)/2}(k_x^2)^{\frac{n+3}{4}}K_{\frac{n+1}{2}}\left(|t-t'|\sqrt{k_x^2}\right),
\end{aligned}
\tag{S10}
$$

where K_η's are modified Bessel functions of the second kind. Plugging the results of Eqs. (S9) and (S10) into Eq. (S8), we see that the poles disappear. Moreover, by imposing the constraint on the y-component ($y=y'=0$), we observe that all the n-even contributions vanish, except $n=2$. For $n=2$, Eq. (S8) becomes

$$
\frac{1}{2(2\pi)^2}\int_{-\infty}^{\infty}dk_x e^{ik_x(x-x')}\frac{e^{-|t-t'|\sqrt{k_x^2}}}{|t-t'|}=\frac{1}{4\pi^2}\frac{1}{|t-t'|^2+|x-x'|^2}.
\tag{S11}
$$

Hence, summing the results of Eqs. (S7) and (S11), we have

$$
\left[\frac{1}{(-\Box)}\right]_{**}=\frac{1}{2\pi}\delta(x-x')\delta(t-t')+\frac{1}{4\pi^2}\frac{1}{(t-t')^2+(x-x')^2},
\tag{S12}
$$

where the symbol $**$ means that we took both the $y-$ and the $z-$coordinate constraints into account. Interestingly, the Fourier transform of the second term in Eq. (S12) is actually

$$
\frac{1}{(t-t')^2+(x-x')^2}=\int\frac{dk_x}{2\pi}\int\frac{d\omega}{2\pi}\frac{e^{ik_x(x-x')+i\omega(t-t')}}{\omega^2+k_x^2}\equiv\frac{1}{\Box_{1+1}},
\tag{S13}
$$

which then yields an effective interaction composed of a sum of a local and a non-local term, i.e.

$$
\left[\frac{1}{(-\Box)}\right]_{**}=\frac{1}{2\pi}\delta(x-x')\delta(t-t')+\frac{1}{4\pi^2}\frac{1}{\Box_{1+1}}.
\tag{S14}
$$

II. EFFECTIVE ACTION AND 1+1-DIMENSIONAL LAGRANGIAN

From the result found in Eq. (S14), the effective action reads

$$\begin{aligned} S_{\rm int} &= -\frac{e^2}{2\varepsilon_0 c}\int d^4r d^4r' j^\mu_{3+1}(r)\frac{1}{(-\Box)}j^{3+1}_\mu(r') \\ &= -\frac{e^2}{2\varepsilon_0 c}\int d^2r d^2r' j^\mu_{1+1}(r)\left[\frac{1}{(-\Box)}\right]_{**} j^{1+1}_\mu(r') \\ &= \underbrace{-\frac{e^2}{4\pi\varepsilon_0 c}\int d^2r j^\mu_{1+1}(r) j^\mu_{1+1}(r)}_{S^1_{\rm int}} \underbrace{-\frac{e^2}{8\pi^2\varepsilon_0 c}\int d^2r d^2r' j^\mu_{1+1}(r)\frac{1}{\Box_{1+1}}j^{1+1}_\mu(r')}_{S^2_{\rm int}}. \end{aligned} \tag{S15}$$

Now, the effective theory has the following partition function

$$\mathcal{Z} = \int \mathcal{D}\bar\psi \int \mathcal{D}\psi \exp\left[\frac{i}{\hbar}(S_{\rm free} + S^1_{\rm int} + S^2_{\rm int})\right], \tag{S16}$$

where we split the effective action in Eq. (S15) in two parts, i.e., $S_{\rm int} = S^1_{\rm int} + S^2_{\rm int}$, and $S_{\rm free}$ contains the *free* Dirac Lagrangian in (1+1)D. Because both interaction terms $S^a_{\rm int}$ (with $a = 1, 2$) are quadratic in the fields, by using a Hubbard-Stratonovich transformation

$$\exp\left\{\frac{i}{\hbar}S^a_{\rm int}[\bar\psi,\psi]\right\} = \int \mathcal{D}\mathcal{A}^a_\mu \exp\left\{\frac{i}{\hbar}\tilde S^a_{\rm int}[\bar\psi,\psi,\mathcal{A}^a_\mu]\right\}, \tag{S17}$$

we introduce auxiliary gauge-fields $\mathcal{A}^a_\mu$ and unveil the underlying gauge theory that mediates the four-fermion (local and non-local) interaction. Thus, the partition function becomes

$$\mathcal{Z} = \int \mathcal{D}\mathcal{A}^1_\mu \int \mathcal{D}\mathcal{A}^2_\mu \int \mathcal{D}\bar\psi \int \mathcal{D}\psi \exp\left\{\frac{i}{\hbar}\int d^2\mathbf{r}\mathcal{L}_{1+1}[\bar\psi,\psi,\mathcal{A}^1_\mu,\mathcal{A}^2_\mu]\right\}, \tag{S18}$$

where

$$\mathcal{L}_{1+1} = i\hbar\bar\psi\gamma^\mu\partial_\mu\psi - ej^\mu\mathcal{A}^1_\mu - \bar e j^\mu\mathcal{A}^2_\mu - g_1 F^1_{\mu\nu}\frac{1}{\Box_{1+1}}F^{\mu\nu}_1 - g_2 F^2_{\mu\nu}F^{\mu\nu}_2, \tag{S19}$$

with $g_1 = \pi\varepsilon_0 c/2$ and $g_2 = \pi^2\varepsilon_0 c$ dimensionless constants. By integrating out the $\mathcal{A}^1_\mu$-field we obtain the Thirring model [2], whereas the Lagrangian for $\mathcal{A}^2_\mu$ gives us the Schwinger model [3]. Both models are exactly solvable in 1+1-dimensions. Notice that $\bar e$ is a dimensionful bare constant, which is in agreement with the Schwinger model. Because the action is dimensionless, all the terms inside the parenthesis in Eq. (9) of the main text must have dimension of (mass)^2 = (length)$^{-2}$. Thus, the product $c_1 A^1_\mu$ and $c_2 A^2_\mu$ (with c_1 and c_2 coupling constants) share the same dimensionality, i.e. (length)$^{-1}$. However, the fields A^1_μ and A^2_μ have different kinematical terms, yielding a different dimensionality to each of them. While A^1_μ has dimension (length)$^{-1}$, leading to a dimensionless coupling $c_1 = e$; A^2_μ is dimensionless and its coupling with the fermionic current has dimension (length)$^{-1}$, i.e. $c_2 = e\Lambda \equiv \bar e$.

The correspondence between Eqs. (S15) and (S19) can be seen explicitly by squaring the gauge fields $\mathcal{A}^1_\mu$ and $\mathcal{A}^2_\mu$. In this manner, we obtain the following effective interactions between the matter currents

$$g_1\left(-F^1_{\mu\nu}\frac{1}{\Box_{1+1}}F^{\mu\nu}_1 - \frac{ej^\mu\mathcal{A}^1_\mu}{g_1}\right) = 2g_1\left(\mathcal{A}^1_\mu\mathcal{A}^\mu_1 - \frac{ej^\mu\mathcal{A}^1_\mu}{2g_1}\right) = 2g_1\left(\mathcal{A}^1_\mu\mathcal{A}^\mu_1 - \frac{2ej^\mu\mathcal{A}^1_\mu}{4g_1} + \frac{e^2 j^\mu j_\mu}{16g_1^2}\right) - \frac{e^2 j^\mu j_\mu}{8g_1},$$

and

$$g_2\left(-F^2_{\mu\nu}F^{\mu\nu}_2 - \frac{\bar e j^\mu\mathcal{A}^2_\mu}{g_2}\right) = 2g_2\left(\mathcal{A}^2_\mu\partial^\nu\partial_\nu\mathcal{A}^\mu_2 - \frac{\bar e j^\mu\mathcal{A}^2_\mu}{2g_2}\right) = 2g_2\left(\mathcal{A}^2_\mu\partial^2\mathcal{A}^\mu_2 - \frac{2\bar e j^\mu\mathcal{A}^2_\mu}{4g_2} + \frac{\bar e^2 j^\mu\partial^{-2}j_\mu}{16g_2^2}\right) - \frac{\bar e^2}{8g_2}j^\mu\frac{1}{\Box_{1+1}}j_\mu,$$

which for $g_1 = \pi\varepsilon_0 c/2$ and $g_2 = \pi^2\varepsilon_0 c$ reproduce those two terms in Eq. (S15).

III. MASSES IN THE THIRRING MODEL AND THE BACKSCATTERING INTERACTION

To investigate the properties of the edge states in presence of an external Zeeman field, which breaks time-reversal symmetry, one may add a mass (i.e. $m\bar{\psi}\psi$) to the Dirac fermions in Eq. (1) of the main text. The parameter m is proportional to the intensity of the Zeeman field, which we consider for simplicity constant in modulus and direction. Because this mass term is not affected by the dimensional-reduction procedure, it also appears in the Thirring model, generating a gap in the boundary modes. In the Hamiltonian picture, this massive term is written as

$$H_m = m \int dx \left(\psi_R^\dagger \psi_L + \psi_L^\dagger \psi_R \right) . \tag{S20}$$

Now, by using the bosonization rules with the Klein factors properly defined [4], the above massive term becomes

$$H_m^{\rm bos} = \frac{m}{\pi} \int dx \cos\left(\sqrt{8\pi} \varphi \right) . \tag{S21}$$

For the bosonic representation, this cosine term, when localized in a small region, acts as a boundary in the system, changing the fermionic orientation of propagation. The existence of such contribution leads to the study of the renormalization group of the Sine-Gordon model, as already analyzed in Ref. [5]. Note that the above term looks similar to the one obtained in Ref. [6], induced by the Umklapp scattering.

At a theoretical level, another possible massive term is $i\Delta\bar{\psi}\gamma^5\psi$ with $\gamma^5 = \gamma^0\gamma^1$. The coefficient Δ is known as the chiral mass and it adds to the fermionic Hamiltonian the following contribution

$$H_\Delta = i\Delta \int dx \left(\psi_R^\dagger \psi_L - \psi_L^\dagger \psi_R \right) , \tag{S22}$$

which mainly differs from the usual Dirac mass by a factor minus between $\psi_R^\dagger \psi_L$ and its conjugated. The minus sign in Eq. (S22) leads to a bosonized Hamiltonian containing an interaction term that depends on a sine function instead of a cosine, i.e.,

$$H_\Delta^{\rm bos} = -\frac{\Delta}{\pi} \int dx \sin\left(\sqrt{8\pi} \varphi \right) . \tag{S23}$$

Here, we can easily recover the standard potential in Eq. (S21) after a constant shift of the scalar field, i.e. $\varphi \to \varphi - \pi/2$.

[1] E. C. Marino, Nucl. Phys. B **408**, 551 (1993).
[2] W. Thirring, Ann. Phys. **3**, 91 (1958).
[3] J. S. Schwinger, Phys. Rev. **128**, 2425 (1962).
[4] T. Lee, J. Korean Phys. Soc. **68**, 1272 (2016).
[5] J. Maciejko, C. Liu, Y. Oreg, X.-L. Qi, C. Wu and S.-C. Zhang, Phys. Rev. Lett. **102**, 256803 (2009).
[6] C. Wu, B. A. Bernevig and S.-C. Zhang, Phys. Rev. Lett. **96**, 106401 (2006).
